# Monoubiquitination of Ancient Ubiquitous Protein 1 Promotes Lipid Droplet Clustering

**DOI:** 10.1371/journal.pone.0072453

**Published:** 2013-09-05

**Authors:** Daniel Lohmann, Johanna Spandl, Ana Stevanovic, Mario Schoene, Julia Philippou-Massier, Christoph Thiele

**Affiliations:** LIMES Life and Medical Sciences Institute, Rheinische Friedrich-Wilhelms-Universität Bonn, Bonn, Germany; The University of New South Wales, Australia

## Abstract

Lipid droplets, the intracellular storage organelles for neutral lipids, exist in a wide range of sizes and of morphologically distinct organization, from loosely dispersed lipid droplets to tightly packed lipid droplet clusters. We show that the lipid droplet protein AUP1 induces cluster formation. A fraction of AUP1 is monoubiquitinated at various lysine residues. This process depends on its internal CUE domain, which is a known ubiquitin-binding domain. AUP1 with a deleted or point mutagenized CUE domain, as well as a lysine-free mutant, are not ubiquitinated and do not induce lipid droplet clustering. When such ubiquitination deficient mutants are fused to ubiquitin, clustering is restored. AUP1 mutants with defective droplet targeting fail to induce clustering. Also, another lipid droplet protein, NSDHL, with a fused ubiquitin does not induce clustering. The data indicate that monoubiquitinated AUP1 on the lipid droplet surface specifically induces clustering, and suggest a homophilic interaction with a second AUP1 molecule or a heterophilic interaction with another ubiquitin-binding protein.

## Introduction

Lipid droplets (LDs) are neutral lipid storage organelles consisting of a hydrophobic core of mainly triacylglycerides and esterified sterols surrounded by a phospholipid monolayer with a number of embedded and associated proteins. Over the last decades LDs have been intensively studied and LDs are appreciated today as dynamic cellular organelles [1]–[5] with a unique proteome [6].

LDs are motile organelles, being able to move rapidly around the cytoplasm [7], [8]. Motility and intracellular redistribution of LDs depends on an intact microtubule network [7], [9], [10] and the motor proteins dynein [11]–[13] and kinesin-1 [14]. LD motility is important for the reorganization in LD distribution in early *Drosophila* embryogenesis [13], [15], [16] and has been suggested to be important for the exchange of lipids between LDs and distinct cellular compartments [17]–[21]. Even though LDs are usually dispersed throughout the cytosol [4], [5], under certain conditions LDs have been observed to aggregate and form densely packed clusters, consisting of numerous individual LDs [5], [10], [22], [23]. For example, it was shown that FSP27 [22], [24], perilipin 1 [23], [25] and core protein of HCV [10], [26] associate with LDs and promote their clustering. Redistribution of LDs is achieved within 16 h after ectopic expression of FSP27 [22] and up to 72 h in the case of core protein of HCV [10]. For HCV core protein it was shown that the process is dynein-dependent [10].

We previously described AUP1 as a monotopic membrane protein localizing to both, LDs and ER membranes [27], [28]. It was also shown that AUP1 can be ubiquitinated [29], binds the E2 ligase Ube2g2 via a C-terminally located G2BR domain [27], [29] and that the AUP1 CUE domain binds dislocation substrates and components of the ER quality control machinery [29], [30]. Here, we present evidence that AUP1 promotes LD clustering and show that modification of AUP1 by a single ubiquitin moiety is sufficient to induce LD clustering.

## Results

### Knockdown of AUP1 in A431 cells causes declustering of LDs

**Figure 1:**
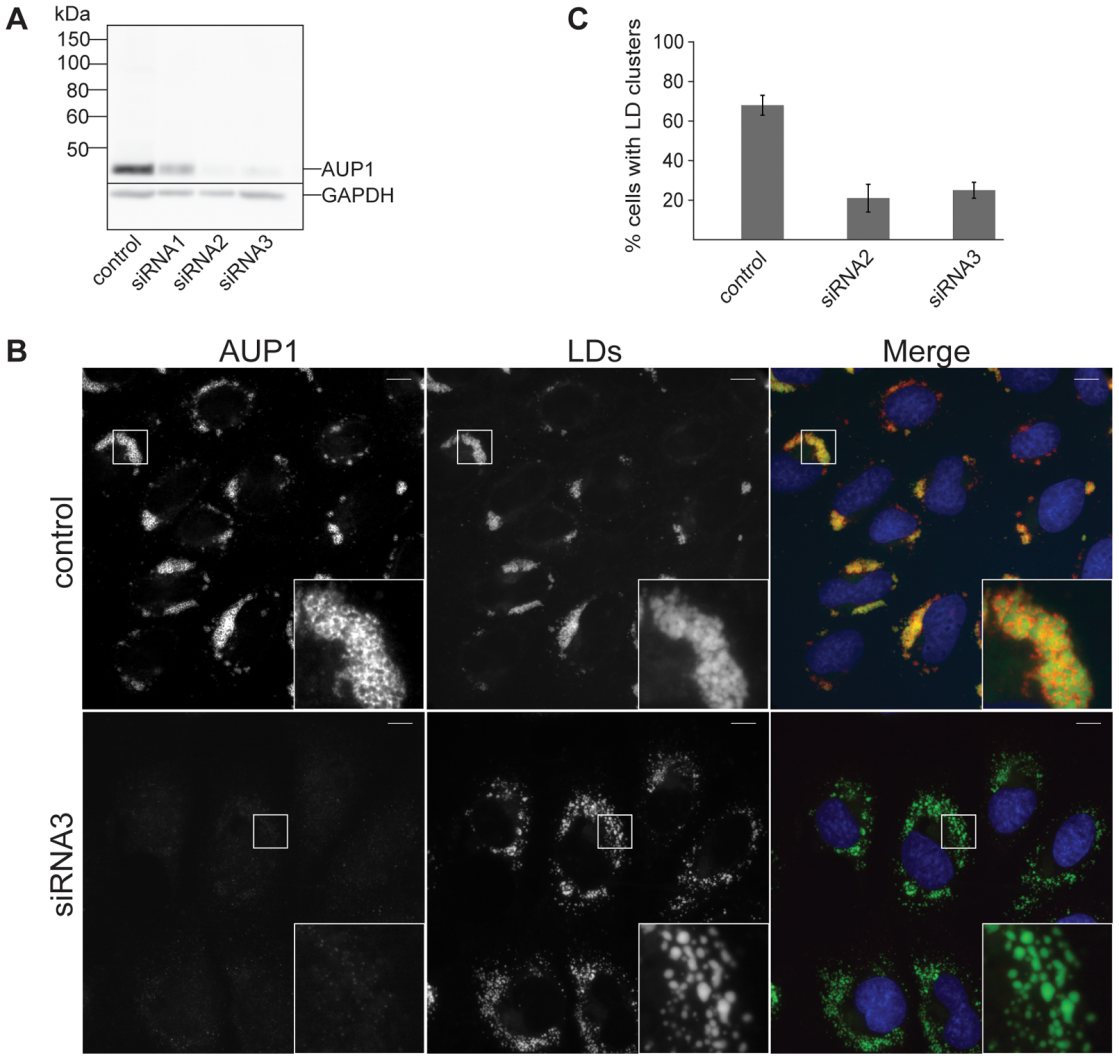
Knockdown of AUP1 causes declustering of LDs. A) A431 cells were either mock transfected or transfected with one of three different siRNAs against AUP1. Cells were lysed and proteins separated by SDS-PAGE and immunoblotted with anti-AUP1 antibody. GAPDH served as loading control. B) Fluorescence micrographs of mock transfected (control) or siRNA treated (siRNA3) A431 cells, both grown in medium supplemented with 50 µM oleate. Cells were immunostained with anti-AUP1 antibody (AUP1, left), and LD540 (LDs, middle panels). Merged images (right) show nuclei stained by DAPI in blue, AUP1 in red and LDs in green. Bars, 10 µm. C) Quantification of LD clustering in mock- (control) or siRNA- (as indicated) treated A431 cells. Results are displayed as average ± standard deviation of three independent experiments. For each individual experiment at least 25 cells were analyzed.

To elucidate the role of AUP1 in the cellular context we analyzed the effect of AUP1 knockdown on the LD phenotype in A431 cells. We analyzed three different stealth siRNAs targeted to different sequences in the AUP1 transcript with respect to their knockdown efficiency. Two of three stealth siRNAs showed a strong reduction of endogenous AUP1 levels in A431 cells (Figure 1A). These two stealth siRNAs were then used to knockdown AUP1 in A431 cells and the LD phenotype was analyzed and compared to mock transfected A431 cells. Knockdown of AUP1 resulted in a striking change in the intracellular distribution of LDs. In mock transfected A431 cells, LDs had a strong tendency to aggregate and form densely packed LD clusters whereas the knockdown of AUP1 strongly reduced LD clustering, and numerous LDs were dispersed throughout the cytoplasm (Figure 1B, C), suggesting that AUP1 plays an active role in the intracellular distribution of LDs. These findings prompted us to look at the role of AUP1 in LD clustering in more detail.

### AUP1 induces LD clustering

**Figure 2:**
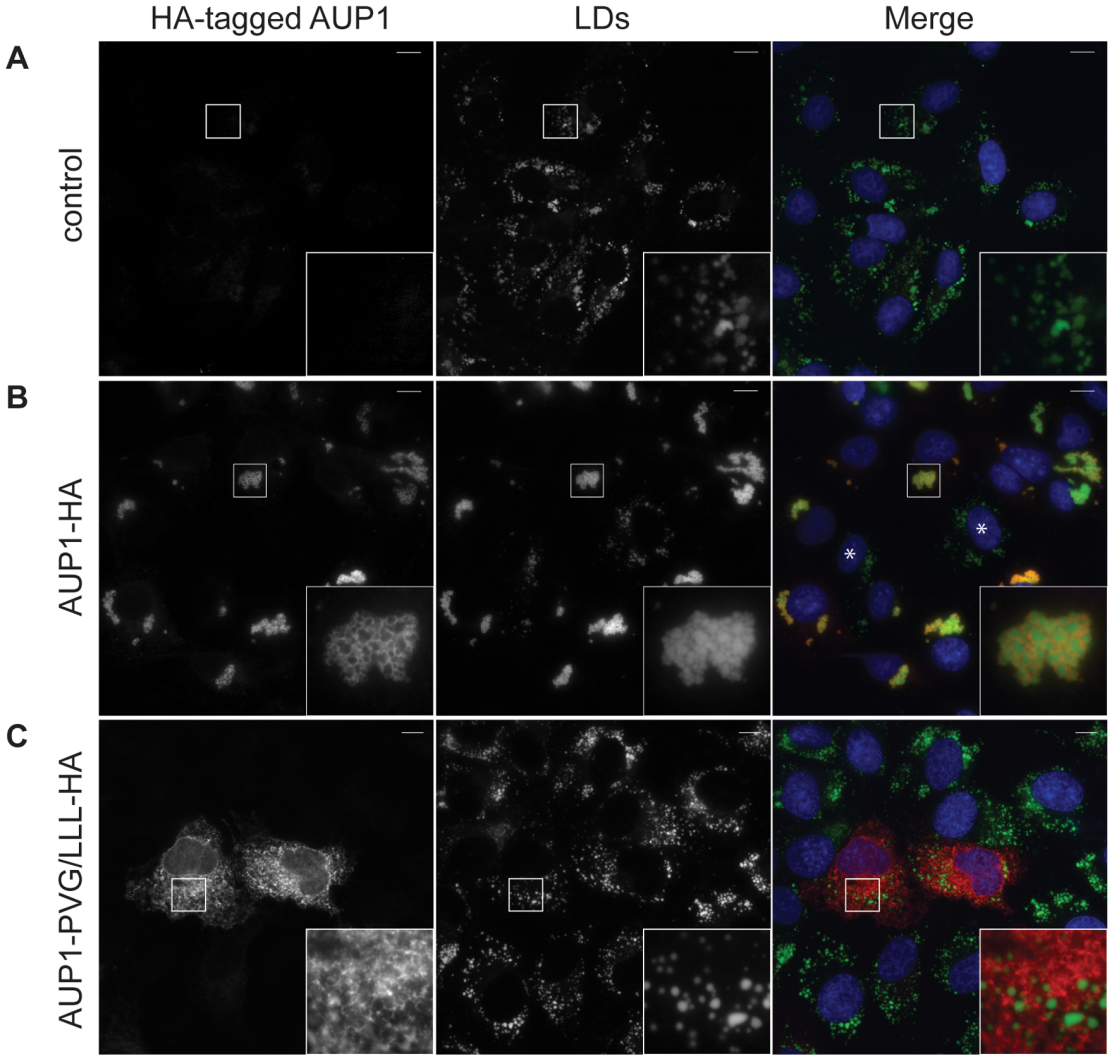
AUP1 overexpression causes LD clustering. A–C) COS7 cells were transfected with empty control vector (control) or different HA-tagged AUP1 constructs as indicated and grown in medium supplemented with 50 µM oleate. Cells were immunostained with anti-HA antibody (left), and LD540 (LDs, middle panels). Merged images (right) show nuclei stained by DAPI in blue, AUP1 in red and LDs in green. Bars, 10 µm. COS7 cells not expressing AUP1-HA do not show LD clustering (marked by asterisk (*)).

**Figure 3:**
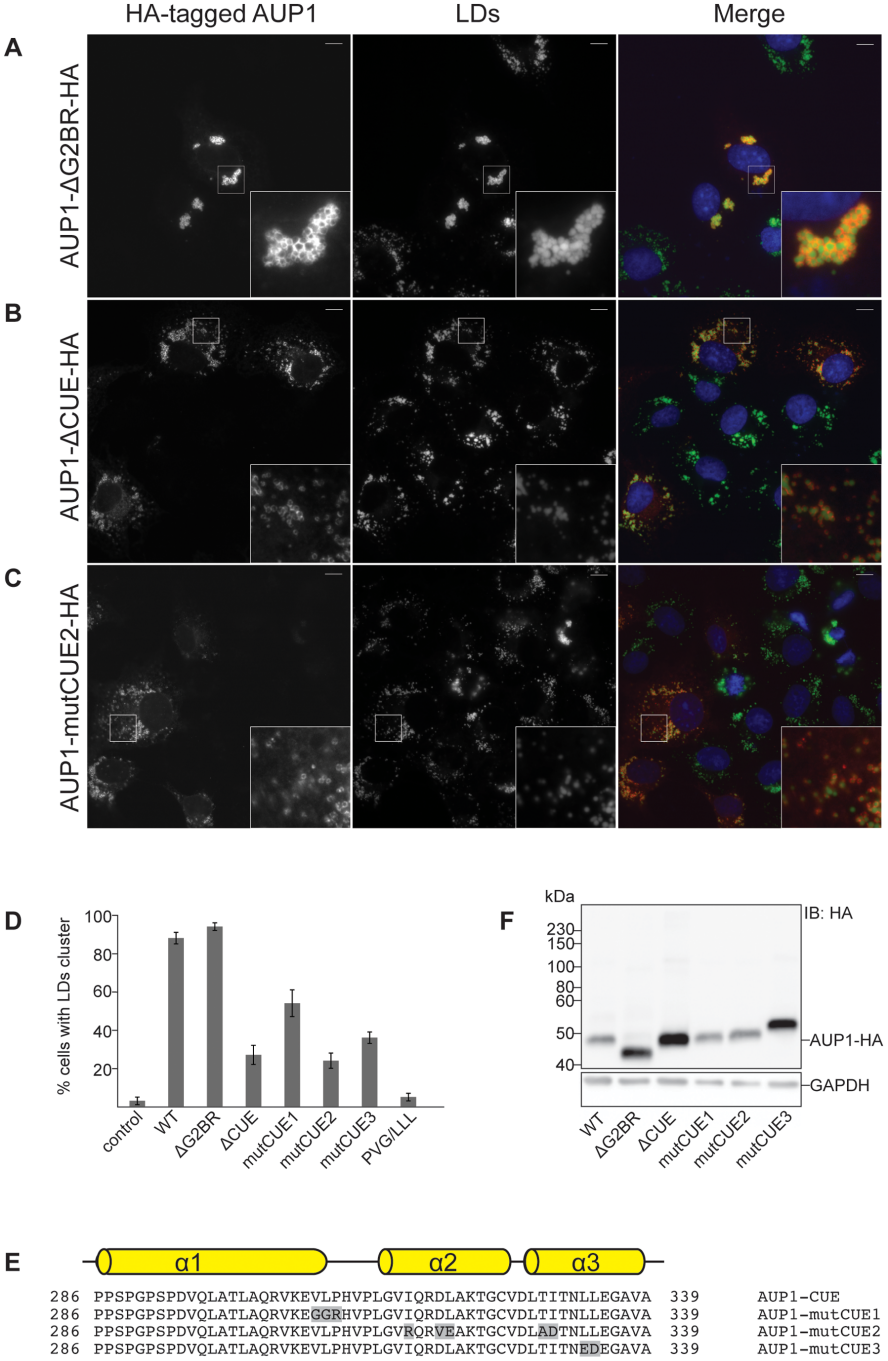
The AUP1 CUE domain is important for LD clustering. A–C) COS7 cells were transfected with different HA-tagged AUP1 domain deletion or mutation constructs as indicated and grown in medium supplemented with 50 µM oleate. Cells were immunostained with anti-HA antibody (left), and LD540 (LDs, middle panels). Merged images (right) show nuclei stained by DAPI in blue, AUP1 in red and LDs in green. Bars, 10 µm. D) Quantification of LD clustering in COS7 cells overexpressing HA-tagged AUP1 constructs as indicated. Empty vector was used as control. Results are displayed as average ± standard deviation of three independent experiments. For each individual experiment at least 25 cells were analyzed. F) Expression levels of HA-tagged AUP1 constructs. Proteins from COS7 cells overexpressing HA-tagged AUP1 constructs as indicated were separated by SDS-PAGE and immunoblotted with anti-HA antibody. GAPDH served as loading control. Note: AUP1-ΔCUE-HA and AUP1-mutCUE3-HA migrated at an apparent molecular weight around five kDa higher than expected. E) Amino acid sequence of the AUP1 CUE domain and predicted relative position of the three α-helices after Prag *et al.* [34]. Mutated amino acid residues of the three AUP1-mutCUE constructs used in this work are highlighted in grey.

For that purpose, we selected a cell line that displays little LD clustering, COS7 fibroblasts, overexpressed different HA-tagged AUP1 constructs, and analyzed the distribution of LDs (Figure 2). COS7 cells transfected with an empty control vector were almost devoid of any LD clusters, and numerous single LDs were dispersed throughout the cytoplasm (Figure 2A). Overexpression of AUP1-HA caused the aggregation of LDs to form one or few clusters consisting of numerous single LDs, whereas the cytoplasm was almost devoid of any single LDs (Figure 2B). Furthermore, as expected from our previous studies, AUP1 localized to these LD clusters (Figure 2B inset). Quantification of clustering (Figure 3D) demonstrates that about 80% of COS7 cells overexpressing AUP1-HA but only 5% of cells transfected with empty control vector showed LD clustering.

Like several other integral LD proteins, AUP1 exhibits a dual distribution in cells, localizing to LDs and the ER. To address the question whether AUP1 localization to LDs is necessary to cause their clustering, we analyzed LD clustering in COS7 cells overexpressing HA-tagged AUP1 with mutations in the LD targeting domain (AUP1-PVG/LLL-HA) that localizes only to the ER and fails to reach LDs [28]. LD clustering in these cells (Figure 2C) is comparable to cells transfected with empty control vector (Figure 3D), demonstrating that AUP1 localization to LDs is necessary to promote their clustering.

### The AUP1 CUE domain is important for LD clustering

To identify functional domains of AUP1 important for LD clustering, we generated HA-tagged truncation and deletion mutants of AUP1 and studied their effect on LD clustering. We focused on two functional domains located at the C-terminus of AUP1, the G2BR and CUE domain. The G2BR domain is essential for binding Ube2g2 [27]. The CUE domain belongs to a group of ubiquitin-binding domains, which have been shown to interact with ubiquitin [31], [32]. Overexpression of AUP1 (1–362) lacking the G2BR domain (AUP1-ΔG2BR-HA) still caused clustering of LDs (Figure 3A) in about 85% of cells analyzed (Figure 3D).

In contrast, only around 30% of COS7 cells overexpressing an internal deletion mutant of AUP1 (Δ295–339) lacking the CUE domain (AUP1-ΔCUE-HA) showed LD clustering (Figure 3B), suggesting an important role for the CUE domain in LD clustering. The CUE domain consists of 3 alpha-helices with conserved residues for binding ubiquitin on helices 1 and 3, and key residues for the helix 1/helix 2 packing interface on helix 2 [33], [34]. We mutated AUP1 within these conserved regions to hinder interaction with ubiquitin. Figure 3E shows the AUP1 CUE mutants generated. Only 30–50% of analyzed COS7 cells overexpressing the different AUP1 CUE mutants showed LD clustering (Figure 3C,D), confirming the importance of the CUE domain for LD clustering. Importantly, truncation of the G2BR domain and deletion or mutation of the CUE domain did not change the localization of AUP1 to LDs (Figure 3A-C inset). To analyze whether different expression levels of the constructs might be responsible for their differing ability to induce LD clustering, the expression levels were analyzed by immunoblotting against the HA-tag, using GAPDH as loading control (Figure 3F). Although the expression levels varied between the different constructs, no apparent correlation between expression levels and LD clustering could be observed.

### AUP1 is ubiquitinated in COS7 cells

Several proteins containing ubiquitin-binding domains have been shown to be modified by ubiquitin themselves [35]–[37]. It was shown in HeLa cells that AUP1 is ubiquitinated and that the integrity of the CUE domain is essential for this process [29].

**Figure 4:**
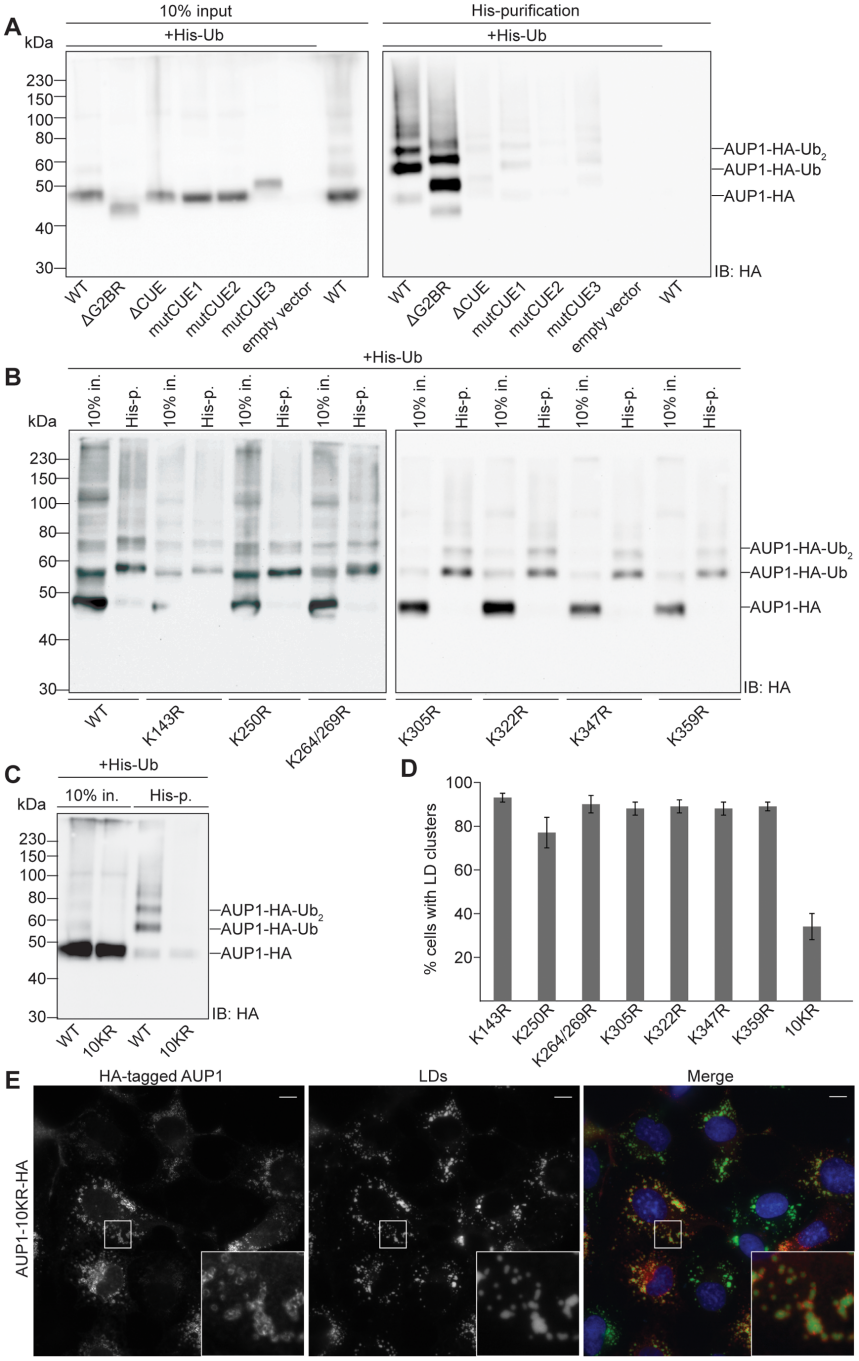
AUP1 is ubiquitinated. A) COS7 cells were transfected with His-ubiquitin and HA-tagged AUP1 constructs and controls (as indicated). His-ubiquitin and His-ubiquitin modified proteins were isolated from cell lysates using Ni-NTA agarose. Proteins from lysates (10% input, left panel) and His-purifications (right panel) were separated by SDS-PAGE and immunoblotted with anti-HA antibody. B, C) COS7 cells were transfected with His-ubiquitin and HA-tagged AUP1 constructs (as indicated). Samples were processed as under A). D) Quantification of LD clustering in COS7 cells overexpressing HA-tagged Lys to Arg mutation AUP1 constructs (as indicated). Results are displayed as average ± standard deviation of three independent experiments. For each individual experiment at least 25 cells were analyzed. E) COS7 cells were transfected with a construct expressing AUP1-10KR-HA and grown in medium supplemented with 50 µM oleate. Cells were immunostained with anti-HA antibody (left), and LD540 (LDs, middle panel). The merged image (right) shows nuclei stained by DAPI in blue, AUP1 in red and LDs in green. Bars, 10 µm.

To analyze if AUP1 is also ubiquitinated in COS7 cells, cells were double transfected with His-ubiquitin and AUP1-HA followed by solubilization with 6 M guanidinium hydrochloride. His-ubiquitin and any His-ubiquitin modified proteins were recovered from cell lysate by binding to Ni-NTA agarose and immunoblotted against the HA-tag of AUP1. Besides some higher molecular weight forms, two major bands for AUP1-HA were detected (Figure 4A, right panel), migrating at an apparent molecular weight of 8 and 16 kDa above the molecular weight for AUP1-HA (Figure 4A, left panel). This molecular weight shift suggests a modification of AUP1 by one or two ubiquitin moieties, respectively. The same shift was observed for AUP1-ΔG2BR-HA (Figure 4A). In contrast, ubiquitinated species were almost undetectable for AUP1-ΔCUE-HA or for the HA-tagged AUP1 CUE mutants (Figure 4A), suggesting that in COS7 cells the CUE domain is essential for AUP1 ubiquitination while the G2BR domain is dispensable for AUP1 ubiquitination.

### AUP1 is ubiquitinated on more than one lysine residue

Ubiquitination usually leads to the formation of a covalent bond between ubiquitin and a lysine residue of the target protein [38]. AUP1 contains ten lysine residues representing potential targets for ubiquitination. To investigate which lysine residues are important for ubiquitination, single lysine to arginine mutations were introduced into HA-tagged AUP1 and those constructs were analyzed with respect to their ubiquitination state upon expression in COS7 cells. The two most C-terminally located lysine residues, K377 and K390 were not analyzed this way. Both, K377 and K390 are deleted in the AUP1-ΔG2BR-HA construct, which showed the same ubiquitination pattern as AUP1-HA (Figure 4A), and were therefore not considered to be major targets for ubiquitination.

As shown in Figure 4B, each AUP1 construct containing single lysine to arginine mutations showed the same ubiquitination pattern as AUP1-HA, suggesting that AUP1 can be ubiquitinated on more than one lysine residue. We therefore generated an HA-tagged AUP1 full-length construct in which all ten lysine residues were mutated to arginine (AUP1-10KR-HA) and analyzed this mutant with respect to its ubiquitination pattern. As shown in Figure 4C, ubiquitination was undetectable for AUP1-10KR-HA. These findings are in line with two recent proteome-wide ubiquitination site surveys, which also found AUP1 to be ubiquitinated at multiple lysine residues [39], [40]. Together, these results demonstrate that AUP1 is ubiquitinated on multiple lysine residues and that multiple lysine to arginine mutations are required for the inhibition of AUP1 ubiquitination.

Next, we wanted to examine whether the different lysine to arginine mutations influence the intracellular distribution of LDs. Overexpression of the single AUP1 lysine to arginine mutants in COS7 cells caused clustering of LDs to comparable extents as AUP1-HA (Figure 4D). In contrast, only around 30% of COS7 cells overexpressing AUP1-10KR-HA showed LD clustering (Figure 4D,E). Again, neither single lysine to arginine mutations (not shown) nor the mutation of all ten lysine residues to arginines changed the localization of AUP1 to LDs (Figure 4E inset).

Taken together, results shown so far suggest that the AUP1 CUE domain alone is not sufficient to promote LD clustering. Rather, the CUE domain is necessary for AUP1 ubiquitination, which in turn is necessary to promote LD clustering.

### AUP1 monoubiquitination is sufficient to promote LD clustering

**Figure 5:**
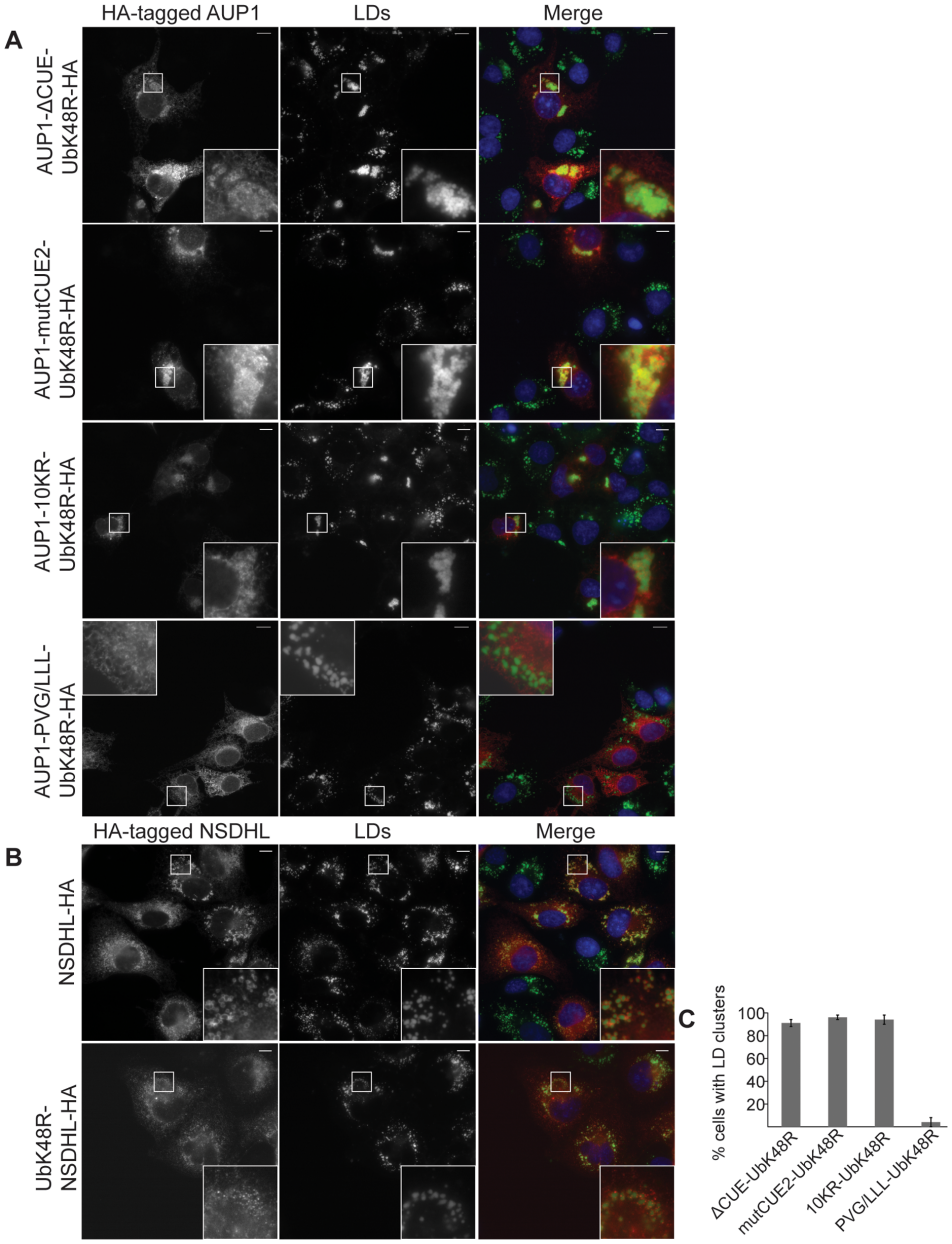
AUP1 monoubiquitination is sufficient to promote LD clustering. A) COS7 cells were transfected with HA-tagged AUP1 mutation constructs (as indicated), fused to monoubiquitin and grown in medium supplemented with 50 µM oleate. Cells were immunostained with anti-HA antibody (left), and LD540 (LDs, middle panels). Merged images (right) show nuclei stained by DAPI in blue, AUP1 in red and LDs in green. Bars, 10 µm. B) COS7 cells were transfected with HA-tagged NSDHL with or without a fused monoubiquitin as indicated and grown in medium supplemented with 50 µM oleate. Cells were immunostained with anti-HA antibody (left), and LD540 (LDs, middle panels). Merged images (right) show nuclei stained by DAPI in blue, AUP1 in red and LDs in green. Bars, 10 µm. C) Quantification of LD clustering in COS7 cells overexpressing HA-tagged AUP1 fusion constructs (as indicated). Results are displayed as average ± standard deviation of three independent experiments. For each individual experiment at least 25 cells were analyzed.

In order to address the question whether AUP1 monoubiquitination alone is sufficient to promote LD clustering, even in the absence of a functional CUE domain, we C-terminally fused HA-tagged ubiquitin to AUP1-ΔCUE (AUP1-ΔCUE-UbK48R-HA, Figure 5A 1^st^ row), AUP1-mutCUE2 (AUP1-mutCUE2-UbK48R-HA, Figure 5A 2^nd^ row) and AUP1-10KR (AUP1-10KR-UbK48R-HA, Figure 5A 3^rd^ row). The ubiquitin fused to AUP1 contained point mutation lysine 48 to arginine (UbK48R) to prevent further polyubiquitination of the chimeric proteins [38]. Overexpression of these chimeric proteins in COS7 cells induced LD clustering to comparable extents as AUP1-HA, with around 90% of cells showing LD clustering (Figure 5C). We conclude that modification of AUP1 by one single ubiquitin moiety is sufficient to induce LD clustering.

Again, LD localization of the chimeric proteins is necessary for clustering, as demonstrated by a construct with the mutated LD targeting domain, which failed to cluster LDs despite the presence of a fused HA-tagged ubiquitin (AUP1-PVG/LLL-UbK48R-HA, Figure 5A 4^th^ row).

Also, when UbK48R was fused to HA-tagged LD protein NSDHL [41] (UbK48R-NSDHL-HA), the chimeric protein retained partial LD localization but failed to cluster LDs (Figure 5B).

## Discussion

Clustering of lipid droplets is a widespread phenomenon that has been observed in a diverse set of eukaryotic cell types and experimental conditions. The yeast *Saccharomyces cerevisiae* is a well-established model organism for the study of lipid metabolism; while yeast LDs are normally spread evenly over the cell, with some preference for contacts with the ER, mutants of the protein FLD1 show dense clusters of LDs with strong propensity for fusion to large LDs [42]–[44]. Also, inhibition of sterol biosynthesis with concomitant accumulation of the precursor squalene leads to LD clustering in yeast [45]. Interestingly, in both cases mammalian cells show similar phenotypes upon mutation or inhibition of the respective orthologous proteins [45], [46], indicating conservation of the unknown underlying mechanisms. In a recent study in Drosophila melanogaster, overexpression of a GFP-tagged putative hydrolase also lead to formation of LD clusters [47].

Several observations regarding LD clustering have also been made in mammalian cells. HCV core protein induces redistribution of LDs to a perinuclear position around the microtubule organizing centers [10], likely caused by enhanced coupling of LDs to microtubule dependent transport. Although morphologically similar to the observations in the present study, the HCV core-induced clusters do not appear to become as dense as AUP1-induced clusters and probably are caused by a different underlying mechanism.

Ectopic expression of perilipin 1 in 3T3-L1 pre-adipocytes causes LD clustering [25], [48], which is reversed upon lipolytic stimulation of these cells [25]. Perilipin 1 itself is phosphorylated during this event, and mutation of one single phosphorylation site within perilipin 1, serine 492, is sufficient to prevent the declustering of LDs upon lipolytic stimulation [25]. Yet, direct evidence that phosphorylation of perilipin 1 at serine 492 is sufficient to drive the dispersion of LDs is lacking, since substitution of serine 492 for negatively charged glutamic acid, mimicking the phosphorylated state of serine 492, is not sufficient to drive the dispersion of LD clusters [25]. Regarding the driving force of the process, a recent study suggested that levels of free fatty acids are a major regulator of the LD remodeling during lipogenesis and lipolysis [49]. A very recent paper demonstrated the importance of cytoskeletal elements and the actin binding protein moesin for perilipin 1 dependent clustering in adipocytes [50].

FSP27 induces LD contact sites that enable transfer of TAG between LDs [24], regulated by perilipin 1 [51], a process important for the formation of large unilocular droplets [52]. FSP27 overexpression causes formation of LD clusters with subsequent formation of large LDs, presumably by lipid transfer [22]. These clusters are morphologically very similar to the AUP1-induced clusters. Similar to AUP1, only FSP27 that localizes to LDs, but not mutants with disrupted LD targeting, is able to induce clustering [22].

The present study provides evidence for a role of LD associated AUP1 in the intracellular distribution of LDs. Mutational analysis revealed an important function for the AUP1 CUE domain in the clustering of LDs, whereas the G2BR domain is dispensable for this process. The function of the CUE domain is best studied for Vps9, a yeast Rab protein guanine nucleotide exchange factor [53]. Work from several laboratories has shown that the CUE domain binds to ubiquitin [34] and promotes intramolecular monoubiquitination of Vps9 by the ubiquitin ligase Rsp5 [32]. Consistent with these observations, we show that AUP1 is predominantly modified by one or two ubiquitin moieties and that this ubiquitination depends on the integrity of the CUE domain. Recently, the same observation has also been made in a different cell line [29]. By mutational analysis we show that AUP1 is ubiquitinated at multiple lysine residues. Although AUP1 lacking ubiquitination still localized efficiently to LDs, its ability to promote LD clustering was strongly reduced. Finally, this study presents direct evidence that modification of AUP1 by one single ubiquitin moiety is sufficient to induce LD clustering.

**Figure 6:**
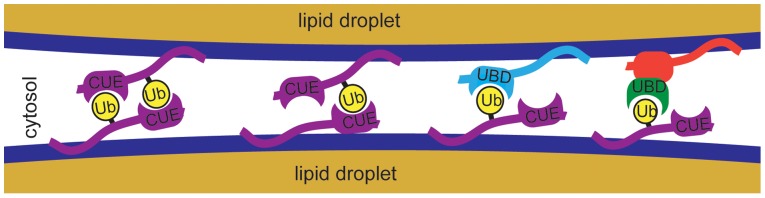
Possible mechanisms for AUP1 induced LD clustering. From left to right: Type 1: monoubiquitinated AUP1 dimerizes *in trans* with another (1A) ubiquitinated or (1B) non-ubiquitinated AUP1 by binding between the ubiquitin moieties and the CUE domains. Type 2: Monoubiquitinated AUP1 interacts *in trans* with another LD protein containing a ubiquitin-binding domain (UBD). Type 3: Interaction of type 1 or 2, but mediated by a soluble adaptor protein.

The mechanism, by which monoubiquitinated AUP1 induces clustering, is not clear. There are several possibilities ranging from very direct interaction to indirect complex pathways (Figure 6); Type 1: monoubiquitinated AUP1 dimerizes *in trans* with another (1A) ubiquitinated or (1B) non-ubiquitinated AUP1 by binding between the ubiquitin moieties and the CUE domains. Type 2: Monoubiquitinated AUP1 interacts *in trans* with another LD protein containing a ubiquitin-binding domain. Type 3: Interaction of type 1 or 2, but mediated by a soluble adaptor protein. Type 4: Indirect action by modulation of other interacting proteins.

An interaction of type 1A would offer the possibility of tight binding because one pair of monoubiquitinated AUP1 could be engaged in two binding events between the ubiquitin and the CUE domain. The fact that AUP1-ΔCUE-UbK48R-HA, lacking a CUE domain, still clusters LDs indicates that type 1A is not absolutely required, but it does neither exclude 1A nor 1B, because of the presence of endogenous untagged AUP1 that may bind to AUP1-ΔCUE-UbK48R-HA resulting in a type 1B interaction.

A type 2 binding to another unknown component would be consistent with all data in this study. A candidate protein with a well-known ubiquitin-binding domain would be Ubxd8, which was identified as a LD component in several studies [17], [54]–[56]. Ubxd8 was already identified as a possible interaction partner of AUP1 [57], but these interactions would likely be *in cis*, not *in trans*. A type 2 interaction *in trans* with a specific LD component would explain another observation that is difficult to reconcile otherwise, which is the fact that AUP1 clusters LDs but, as far as we could observe by standard fluorescence microscopy, does not lead to formation of condensed or clustered structures of its other target organelle, the ER.

One can exclude a type 3 interaction, mediated by a soluble adaptor, at least as a general monoubiquitin-crosslinking adaptor, because such an adaptor would also bridge UbK48R-NSDHL-HA and induce LD clustering. One would have to postulate that such an adaptor binds only to monoubiquitinated AUP1 but not to other proteins.

Due to their undefined nature, type 4 interactions are subject to speculation. The most substantiated one would be a modulation of FSP27-dependent lipid droplet clustering [22], [51], possibly by interference with the ubiquitin-dependent degradation of FSP27 [58].

Noteworthy, recruitment of cytosolic p62/SQSTM1 to ubiquitinated mitochondria has been observed to promote the clustering of these mitochondria [59], [60]. P62/SQSTM1 itself contains a ubiquitin-binding domain, which has been shown to bind ubiquitin [61], [62]. Furthermore, it has been shown that p62/SQSTM1 self-oligomerizes [63] and that mutation of amino acid residues of p62/SQSTM1 important for oligomerization attenuates the clustering of mitochondria [60]. Hence, it has been proposed that oligomerized p62/SQSTM1 can simultaneously bind several ubiquitinated mitochondrial proteins via their ubiquitin-binding domains and thereby tether individual mitochondria [60].

Future elucidation of the precise mechanism of cluster formation by monoubiquitinated AUP1 will contribute to understanding the enigmatic physiological function of LD clustering.

## Materials and Methods

### Antibodies

The following antibodies were used: anti-AUP1 [27], anti-HA (clone F-7, Santa Cruz Biotechnology), anti-GAPDH (NovusBiologicals, NB300–221), Alexa488-conjugated secondary antibody (Invitrogen) and HRP-coupled secondary antibodies (Jackson ImmunoResearch).

### Cell Culture

COS7 cells (*Cercopithecus aethiops*, from ATCC®, Number: CRL-1651) and A431 cells (*Homo Sapiens*, from ATCC®, Number: CRL-1555) were cultured in DMEM (Gibco, 41965) supplemented with 10% FCS (Gibco, 10437) and maintained in a humidified incubator at 37°C and 5% CO_2_. OPTI-MEM (Gibco, 11058).

### DNA constructs

DNA sequences with various mutations, deletions or insertions were constructed using standard cloning techniques and cloned into 3HA expression vectors. All constructs were verified by sequencing. For details see Table S1.

### Knockdown of AUP1

The following siRNA sequence pairs were used:

siRNA1 (Invitrogen, HSS141340)

3′-CAUCACCAAGGGAACUCAGUCCCUA-5′; 5′-UAGGGACUGAGUUCCCU

UGGUGAUG-3′

siRNA2 (Invitrogen, HSS141341)

3′-GGAGCGCAAGCAAGCACUAUAUGAA-5′; 5′-UUCAUAUAGUGCUUGCU

UGCGCUCC-3′

siRNA3 (Invitrogen, HSS182853)

3′-ACAGCCCUAACAUUUGCCAAGUCUU-5′; 5′-AAGACUUGGCAAAUGUUA

GGGCUGU-3′

In general the Invitrogen protocols for Lipofectamine 2000 transfection were followed. In detail: Approximately 5000 A431 cells were plated per well of a 24-well plate in 1 ml DMEM +10% FCS the day before transfection. The next day cells were washed with PBS and 200 µl Opti-MEM per well were added. Lipofectamine 2000 (0.8 µl) was mixed with 50 μl Opti-MEM, and 2 µl siRNA (20 µM) was mixed with another 50 µl Opti-MEM. Both mixes were incubated separately for 5 min at RT, combined and incubated for another 20 min. The entire transfection mix was added drop wise to the cells. Cells were incubated over night and then the transfection medium was replaced by 1 ml per well fresh DMEM +10% FCS supplemented with 50 µM oleate. For analysis of cellular proteins or analysis by microscopy cells were harvested or fixed 72 h post transfection.

### Transfection of COS7 cells for microscopy, protein expression levels and ubiquitination assay

In general the Invitrogen protocols for Lipofectamine 2000 transfection were followed. In detail: For transfection in 24-well (microscopy and protein expression levels) and 6-well (ubiquitination assay) plates, cells were grown until reaching a confluency of around 60–70%. Cells were washed with PBS and Opti-MEM were added (200 µl to each well of a 24-well or 1 ml to each well of a 6-well plate). Lipofectamine 2000 (3–4 µl) was mixed with 100 µl Opti-MEM, and 1 µg of plasmid DNA was mixed with another 100 µl of Opti-MEM. Both mixes were incubated separately for 5 min at RT, combined and incubated for another 20 min. From this transfection mix, 40 µl per well of a 24-well or 200 µl per well of a 6-well plate were added drop-wise to the cells. 4 h post transfection medium was replaced by 1 ml per well of a 24-well or 3 ml per well of a 6-well plate of fresh DMEM +10% FCS supplemented with 50 µM oleate. Cells were analyzed 24 h post transfection.

### Fluorescence microscopy of fixed samples

Cells were prepared as described. 24 h post transfection cells were washed with PBS and fixed in PBS with 3.7% (w/v) paraformaldehyde. After 30 min paraformaldehyde was removed and cells were washed with PBS. Fixed cells were stored in PBS at 4°C and prepared for fluorescence microscopy the next day. Fixed cells were incubated in blocking buffer (PBS with 1% BSA and 0.2% saponin) for 30 min followed by incubation with primary antibody (HA-F7 antibody diluted 1∶100 and AUP1 antibody diluted 1∶500) in blocking buffer for 1 h. After washing (3×10 min with blocking buffer) cells were incubated with secondary antibody (diluted 1∶500). Cells were washed (3×10 min with blocking buffer) and stained with DAPI (1µg/ml in PBS) and LD540 [8] in PBS for 15 min. Cells were washed 3×10 min with PBS and 1× with dH_2_O and mounted with 5 µl mowiol/DABCO (6 g glycerol, 2.4 g mowiol, 6 ml dH_2_O, 12 ml 0.2 M Tris-HCl pH 8.5, 0.1% DABCO) on microscope slides. Images were acquired with a ZeissAxio Observer.Z1 microscope (Carl Zeiss; Oberkochen, Germany) equipped with a 63×/NA1.4 objective, optovar magnification changer and a Photometrics Coolsnap K4 camera. Light source was a Polychrome V 150 W xenon lamp (TillPhotonics; Graeffeling, Germany). Images were processed using ImageJ (National Institutes of Health).

### Protein expression levels

After washing cells with PBS, cells were lysed directly in 2× Sample Buffer (50 mM Tris-HCl pH 6.8, 1.6% SDS, 0.0016% Bromophenol blue, 8% (w/v) glycerol, 0.8% betamercaptoethanol). After boiling at 95°C for 5–10 min equal amounts were separated on 10% SDS-PAGE gels. Proteins were blotted onto nitrocellulose membranes and the membrane cut just below the 40 kDa signal from the protein ladder. The upper half was immunoblotted with anti-HA antibody (diluted 1∶1000), the lower half with anti-GAPDH antibody (diluted 1∶1000).

### Ubiquitination assay

Cells were plated and transfected as described above using His-tagged ubiquitin (0.5 µg/100 µl Opti-MEM) and HA-tagged AUP1 plasmids, followed by His-tag purification as follows. Cells were washed with PBS and lysed in 1 ml lysis buffer (6 M guanidinium hydrochloride, 100 mM disodium hydrogen phosphate, 0.5% Triton-X100, 20 mM imidazole, 10 mM Tris-HCl pH 7.4). An aliquot of 100 µl of cell lysate was kept for 10% input and subjected to chloroform-methanol protein precipitation [64]. Ni-Agarose (35 µl of 50% slurry of Ni-NTA Superflow, Qiagen) was added to the remaining cell lysate and incubated on a rotary wheel at RT for 2–3 h, centrifuged (1500 g, 1 min, RT) and Ni-Agarose was washed three times with washing buffer (8 M urea, 100 mM disodium hydrogen phosphate, 0.5% Triton-X100, 20 mM imidazole, 10 mM Tris-HCl pH 7.4) followed by elution with 50 µl elution buffer (250 mM imidazole, 150 mM NaCl, 25 mM Tris-HCl pH 6.0). Finally, 40 µl of 5× sample buffer were added and proteins were separated by 10% SDS-PAGE and analyzed by immunoblotting, using anti-HA antibody (diluted 1∶1000).

## Supporting Information

Table S1 **List of primers and DNA constructs.**
(DOCX)
