# Supplementary material for: Monoubiquitination of Ancient Ubiquitous Protein 1 Promotes Lipid Droplet Clustering
Source: PLoS One. 2013 Sep 5;8(9):e72453. doi: 10.1371/journal.pone.0072453 (PMC3764060; doi:10.1371/journal.pone.0072453)
Supplement: Table S1 — List of primers and DNA constructs. (DOCX) [file pone.0072453.s001.docx]

**Table S1. List of primers and DNA constructs**

| Name | Alias | Vector/Resistance | Primer and template or source | Sites |
| --- | --- | --- | --- | --- |
| AUP1-HA | pCT7 | pCDNA3.1Hygro-3HA/amp | hAUP1_HA_Hindfor: CTAGATAAGCTTGCCATGGAGCTTCCCTCAGGGCC; hAUP1_HA_Xba_rev CTATCTAGAGTCAGCCTCCTGGGCTCG;  Template: AUP1-EST; As described in [27] | HindIII/  XbaI |
| AUP1-∆G2BR-HA | pCT15 | pCDNA3.1Hygro-3HA/amp | hAUP1_HA_Hindfor CTAGATAAGCTTGCCATGGAGCTTCCCTCAGGGCC; AUP_trunc2_nostop_xba CTATCTAGAGCTGGGAAACTTGGAGGCAG; Template: pAUP1; As described in [27] | HindIII/  XbaI |
| 6His-Ub | His_Ub |  | Wolfgang Zachariae, MPI of Biochemistry Muenchen |  |
| AUP1-  PVG/LLL-HA | PVG/LLL-HA | pCDNA3.1Hygro-3HA/amp | AUP1-PVG/LLL-HA; As described in [28] | HindIII/  XbaI |
| AUP1-mutCUE1-HA | pDL45 | pCDNA3.1Hygro-3HA/amp | AUP1_CUE_VLP-GGR_for  GCTCAGAGAGTCAAGGAAGGTGGGCGCCATGTGCCATTGGG; AUP1_CUE_VLP-GGR_rev  CCCAATGGCACATGGCGCCCACCTTCCTTGACTCTCTGAGC; AUP1_HindIII_for GTGCCAAGCTTGCCATGGAGC; AUP1_rev  GGATCCTCTAGAGTCAGC; Template: pCT7 | HindIII/  XbaI |
| AUP1_CUE_TI\AD_3HA | pDL64 | pCDNA3.1Hygro-3HA/amp | AUP1_CUE_TI\AD_for GGCTGTGTAGACTTGGCTGACACTAATCTGCTTGAGG; AUP1_CUE_TI\AD_rev  CCTCAAGCAGATTAGTGTCAGCCAAGTCTACACAGCC; AUP1_HindIII_for and AUP1_rev; Template: pCT7 | HindIII/  XbaI |
| AUP1-mutCUE2-HA | pDL71 | pCDNA3.1Hygro-3HA/amp | AUP1_CUE_VIQRDL\VRQRVD_for  CCATGTGCCATTGGGTGTCCGCCAGAGAGTCGAGGCCAAGACTGGCTGTGTAGAC;  AUP1_CUE_VIQRDL\VRQRVD_rev  GTCTACACAGCCAGTCTTGGCCTCGACTCTCTGGCGGACACCCAATGGCACATGG; AUP1_HindIII_for and AUP1_rev; Template: pDL64 | HindIII/  XbaI |
| AUP1-K305R-HA | pDL75 | pCDNA3.1Hygro-3HA/amp | AUP1_K305R_for  GCTCAGAGAGTCCGGGAAGTTTTGCC;  AUP1_K305R_rev  GGCAAAACTTCCCGGACTCTCTGAGC; AUP1_HindIII_for and AUP1_rev; Template: pCT7 | HindIII/  XbaI |
| AUP1-K322R-HA | pDL76 | pCDNA3.1Hygro-3HA/amp | AUP1_K322R_for  CCAGAGAGACCTGGCCCGGACTGGCTGTGTAGACTTG; AUP1_K322R_rev  CAAGTCTACACAGCCAGTCTTGGCCAGGTCTCTCTGG; AUP1_HindIII_for and AUP1_rev; Template: pCT7 | HindIII/  XbaI |
| AUP1-K143R-HA | pDL87 | pCDNA3.1Hygro-3HA/amp | AUP1_K1/R_for  GGTGGAGTCACTCAGGAGATTCTGTGCTTCC;  AUP1_K1/R_rev GGAAGCACAGAATCTCCTGAGTGACTCCACC;  AUP1_HindIII_for and AUP1_rev; Template: pCT7 | HindIII/  XbaI |
| AUP1- K250R-HA | pDL89 | pCDNA3.1Hygro-3HA/amp | AUP1K2Rnew-for  CAGCTGGTGGCCAGGGAATTGG;  AUP1_K2/R_rev  CCCTGTCTGGCCCAATTCCCTGGCCACCAGC;  AUP1_HindIII_for and AUP1_rev; Template: pCT7 | HindIII/  XbaI |
| AUP1-K264R/K269R-HA | pDL91 | pCDNA3.1Hygro-3HA/amp | AUP1_KK3-4/RR_for  CCAGCTGACAGAGCAGAGCACATGAGGCGACAAAGACACCCC;  AUP1_KK3-4/RR_rev  GGGGTGTCTTTGTCGCCTCATGTGCTCTGCTCTGTCAGCTGG; AUP1_HindIII_for and AUP1_rev; Template: pCT7 | HindIII/  XbaI |
| AUP1-K347R-HA | pDL130 | pCDNA3.1Hygro-3HA/amp | AUP1_K347R_for  GCCTGAAGACATCACCCGGGGAACTCAGTCC;  AUP1_K347R_rev  GGACTGAGTTCCCCGGGTGATGTCTTCAGGC;  AUP1_HindIII_for and AUP1_rev; Template: pCT7 | HindIII/  XbaI |
| AUP1-K359R-HA | pDL131 | pCDNA3.1Hygro-3HA/amp | AUP1_K359R_for  GCCTCTGCCTCCCGGTTTCCCAGCTCTGG;  AUP1_K359R_rev CCAGAGCTGGGAAACCGGGAGGCAGAGGC;  AUP1_HindIII_for and AUP1_rev; Template: pCT7 | HindIII/  XbaI |
| AUP1-mutCUE3-HA | pDL174 | pCDNA3.1Hygro-3HA/amp | AUP1_L333E_L334D_for  CTATCACTAATGAGGATGAGGGGGCCG;  AUP1_L333E_L334D_rev  CGGCCCCCTCATCCTCATTAGTGATAG;  AUP1_HindIII_for and AUP1_rev; Template: pCT7 | HindIII/  XbaI |
| Ub-K48R-  noStop | pDL189 | pCDNA3/amp | Ub_K48R_for  GCTGGGCGACAGCTGGAAGATGG; Ub_K48R_rev  CCATCTTCCAGCTGTCGCCCAGC; Ub-EcorI-for  CGGGAATTCTCCATGCAGATCTTCG; Ub-noStop-XbaI-rev GCCTCTAGAACCACCTCTTAG; Template: Ub-3HA | EcoRI/  XbaI |
| AUP1-mutCUE2-UbK48R-HA | pDL198 | pCDNA3.1Hygro-3HA/amp | AUP1_HindIII_for and AUP1_nostop_EcorI_rev  GGTAGAATTCGTCAGCCTCCTGGGCTCG;  Template: pDL71; HindIII/EcoRI cloned into pDL189 HindIII/EcoRI cut again HindIII/XbaI cloned into pCDNA3.1Hygro-3HA HindIII/XbaI | HindIII/  XbaI |
| AUP1_340-410_ | pDL206 | pCDNA3/amp | AUP1-dCUE-for GCCGTAGAATTCATGCCTGAAG;  AUP1_rev; Template: pCT7 | EcoRI/  XbaI |
| AUP1_1-293_ | pDL207 | pCDNA3/amp | AUP1-dCUE-rev GCCAGTTGGAATTCAGGAGAAG;  AUP1_HindIII_for; Template: pCT7 | HindIII/  EcoRI |
| AUP1_Δ295-339_V294E | pDL212 | pCDNA3/amp | pDL206 EcoR/XbaI cloned into pDL207 EcoRI/XbaI | EcoRI/  XbaI |
| AUP1-ΔCUE-HA | pDL213 | pCDNA3.1Hygro-3HA/amp | Source: pDL212 | HindIII/  XbaI |
| NSDHL-HA | pDL247 | pCDNA3.1Hygro-3HA/amp | NSDHLfull-HindIII-for CTTAAGCTTGCCATGGAACCAGCAG;  NSDHLfull-XbaI-rev GGTATCTAGACTTTTTGGCTC; Template: pCT76 | HindIII/  XbaI |
| AUP1-ΔCUE-UbK48R-HA | pDL263 | pCDNA3.1Hygro-3HA/amp | AUP1_HindIII_for and AUP1_nostop_EcorI_rev; Template: pDL213; Sequential digestion with HindIII/EcoRI cloned into pDL189 HindIII/EcoRI cut again HindIII/XbaI cloned into pCDNA3.1Hygro-3HA HindIII/XbaI | HindIII/  XbaI |
| NSDHL_noStop | pDL288 | pCDNA3/amp | NSDHL-EcorI-for CTTGAATTCGCCATGGAACCAGCAG;  NSDHL-XbaI-rev CTCTCTAGACTTGACCCTCCG; Template: pCT76 | EcoRI/  XbaI |
| Ub_K48R_NSDHL_3HA | pDL290 | pCDNA3.1Hygro-3HA/amp | Ub-NoLys-HindIII-for CTGAAGCTTTCCATGCAGATCTTCG; Ub-noLys-EcorI-rev GCCGAATTCACCACCTCTTAG; Template: pDL189; HindIII/EcoRI cloned into pDL288 HindIII/EcoRI cut again HindIII/XbaI cloned into pCDNA3.1Hygro-3HA HindIII/XbaI | HindIII/  XbaI |
| AUP1-10KR-HA | pDL366 | pCDNA3.1Hygro-3HA/amp | AUP1-10KR-HA was generated by sequential mutagenisis of single lysine residues to arginines using pDL91 as template. The following Primers were used in addition to mutagenise K377 and K390: AUP1-K377-for CATTTGCCAGCTCTTCCTGG; AUP1-K377-for  CCAGGAAGAGCTGGCAAATG; AUP1-K390-for  CAGGAGCGCAGGCAAGCAC; AUP1-K390-rev  GTGCTTGCCTGCGCTCCTG; AUP1_HindIII_for and AUP1_rev | HindIII/  XbaI |
| AUP1-10KR-UbK48R-HA | pDL368 | pCDNA3.1Hygro-3HA/amp | AUP1_HindIII_for and AUP1_nostop_EcorI_rev; Template: pDL366; HindIII/EcoRI cloned into pDL189 HindIII/EcoRI cut again HindIII/XbaI cloned into pCDNA3.1Hygro-3HA HindIII/XbaI | HindIII/  XbaI |
| AUP1-PVG/LLL-UbK48R-HA | pDL410 | pCDNA3.1Hygro-3HA/amp | AUP1_HindIII_for and AUP1_nostop_EcorI_rev; Template: AUP1-PVG/LLL-HA; HindIII/EcoRI cloned into pDL189 HindIII/EcoRI cut again HindIII/XbaI cloned into pCDNA3.1Hygro-3HA HindIII/XbaI | HindIII/XbaI |
